# Supplementary material for: Roton-like acoustical dispersion relations in 3D metamaterials
Source: Nat Commun. 2021 Jun 2;12:3278. doi: 10.1038/s41467-021-23574-2 (PMC8172548; doi:10.1038/s41467-021-23574-2)
Supplement: Supplementary file 1 — Supplementary Information [file 41467_2021_23574_MOESM1_ESM.pdf]

**Supplementary Information**

# Roton-like acoustical dispersion relations in 3D metamaterials

Yi Chen[1], Muamer Kadic[2,3], and Martin Wegener[1,2,*]

[1]Institute of Applied Physics, Karlsruhe Institute of Technology (KIT), 76128 Karlsruhe, Germany

[2]Institute of Nanotechnology, Karlsruhe Institute of Technology (KIT), 76128 Karlsruhe, Germany

[3]Institut FEMTO-ST, UMR 6174, CNRS, Université de Bourgogne Franche-Comté, 25000 Besançon, France

*Corresponding author. Email: martin.wegener@kit.edu

## Supplementary Figures

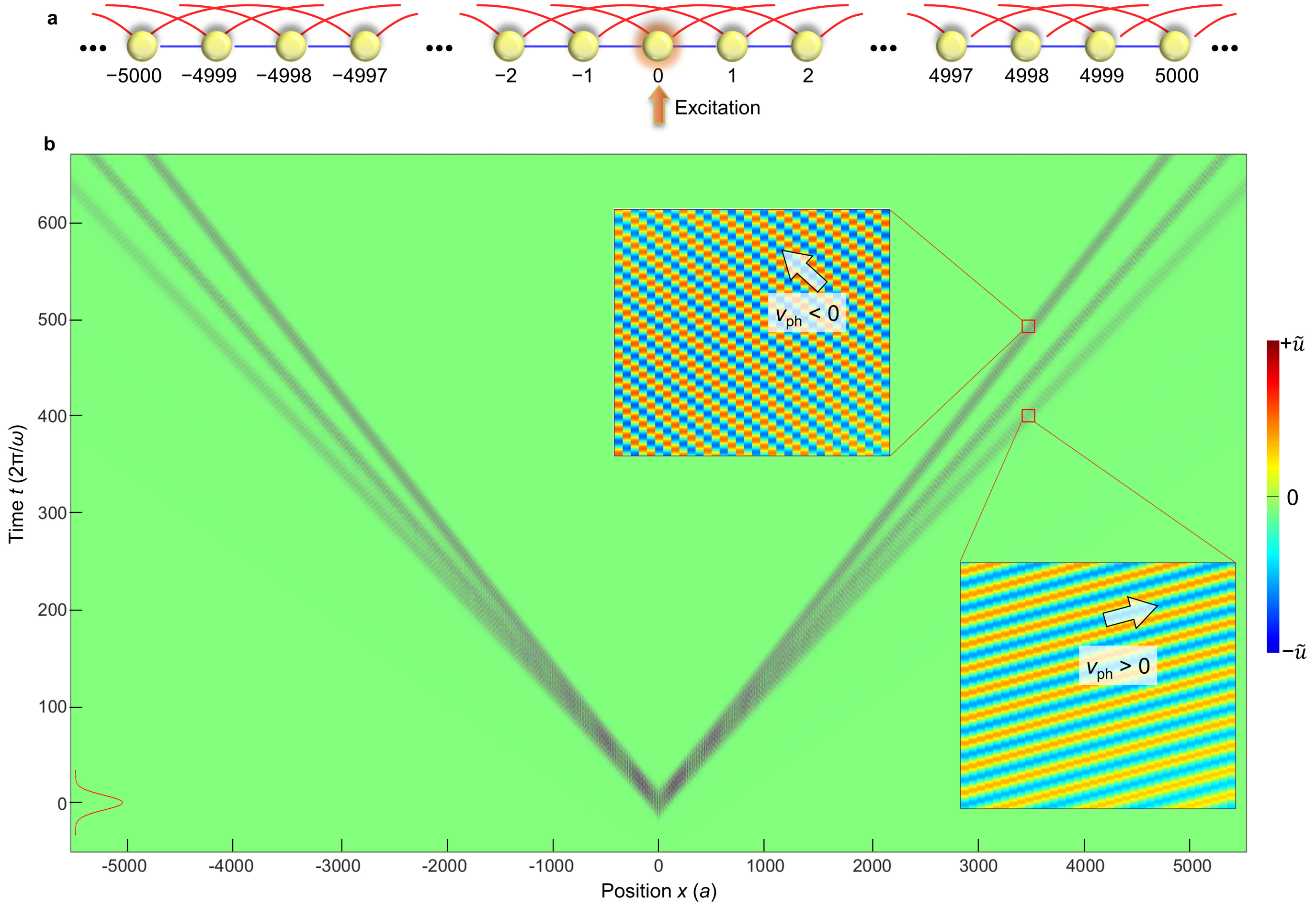


**Supplementary Figure 1 | Wave packet dynamics for the roton-like dispersion.** (a) The displacement of a single mass, $u_0(t)$, of the 1D toy model for $N = 3$ and $K_3/K_1$ =3 (cf. Fig. 1) is driven versus time by a Gaussian pulse with carrier frequency $\omega$, i.e., $u_0(t) = \tilde{u}\cos(\omega t)\exp(-(t/\tau)^2)$ with $\omega = 0.5\omega_0$ and $\tau = 100/\omega$. The Gaussian envelope (red) is shown at the left bottom corner. (b) The response of the chain $u_n(t)$ is exhibited on a false-color scale versus spatial coordinate $x = na$ and time $t$. Two triplets of Gaussian wave packets emerge. One triplet moves to the left, the other one to the right-hand side. Each triplet comprises two forward waves and one backward wave (cf. Fig. 1). The inset shows a magnified view of a snapshot of a backward wave and a forward wave, respectively. The phase velocity can be obtained from the slope of the lines of constant phase. The group velocity can be inferred from the slope of the three wave packets emerging from the excitation location $x = 0$. For the two backward waves, phase and group velocity point in opposite directions.

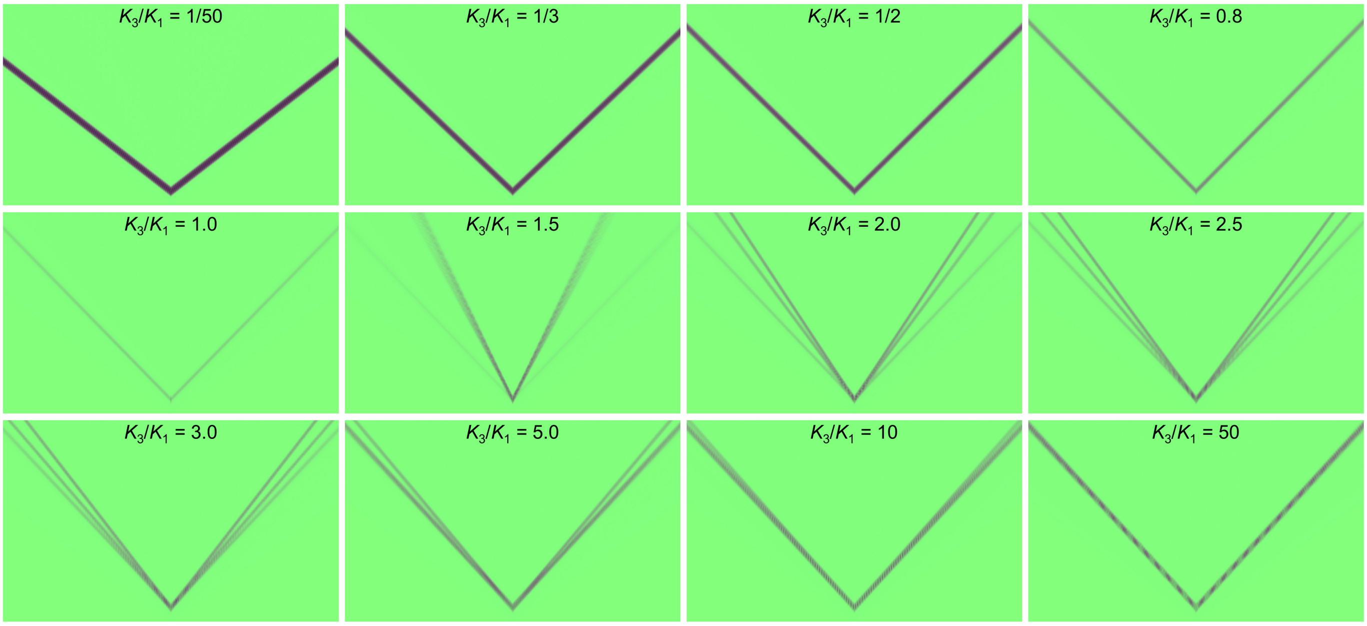


**Supplementary Figure 2 | Parameter variation for wave packet dynamics.** Same as Supplementary Figure 1, but for different ratios of the spring constants in the 1D toy model, $K_3/K_1$, at fixed phase velocity in the limit $k \to 0$, i.e., $v_{\rm ph} = a\sqrt{(K_1 + K_N N^2)/m} = \text{const.}$ (cf. Fig. 2(b)). The scales are the same as in Supplementary Figure 1 and are hence omitted. The triplets of wave packets in Supplementary Figure 1 merge into single wave packets in the two limits $K_3/K_1 \to 0$ (negligible beyond-nearest-neighbor interaction) and $K_3/K_1 \to \infty$ (negligible nearest-neighbor interaction). This behavior is consistent with the expectation based on the dispersion relations shown in Fig. 2.

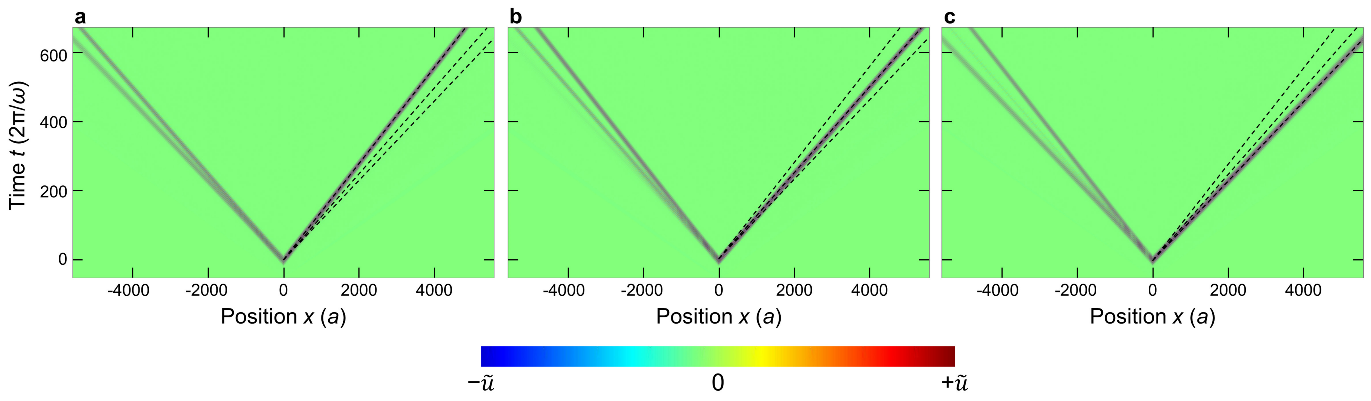


**Supplementary Figure 3 | Controlling the coupling to the right-moving wave packets.** As Supplementary Figure 1, but we do not only excite the center mass at site $n = 0$ as in Supplementary Figure 1 but also its two neighbor masses to the left and right at sites $n = -1$ and $n = +1$, respectively. As a result, only a single wave packet (rather than a triplet as in Supplementary Figure 1) propagates to the right-hand side in each of the three cases (a)-(c). For case (a), only a single wave packet with negative phase velocity propagating to the right-hand side emerges. The three dashed black lines for $x > 0$ in (a)-(c) are guides to the eye and refer to the three wave packets in Supplementary Figure 1. In (a)-(c), we use the excitation conditions $u_0(t) = \tilde{u}_0 \cos(\omega t) \exp(-(t/\tau)^2)$, $u_{-1}(t) = \tilde{u}_{-1} \cos(\omega t + \phi_{-1}) \exp(-(t/\tau)^2)$, and $u_{+1}(t) = \tilde{u}_{+1} \cos(\omega t + \phi_{+1}) \exp(-(t/\tau)^2)$. As in Supplementary Figure 1, we choose $\omega = 0.5\ \omega_0$ and $\tau = 100/\omega$. (a) $\tilde{u}_{-1} = \tilde{u}_{+1} = 0.5\ \tilde{u}_0$, $\phi_{-1} = -\pi/2$, and $\phi_{+1} = +\pi/2$. (b) $\tilde{u}_{-1} = \tilde{u}_{+1} = \tilde{u}_0$, $\phi_{-1} = -0.8\ \pi$, and $\phi_{+1} = +0.8\ \pi$. (c) $\tilde{u}_{-1} = \tilde{u}_{+1} = \tilde{u}_0$, $\phi_{-1} = -0.2\ \pi$, and $\phi_{+1} = +0.2\ \pi$.

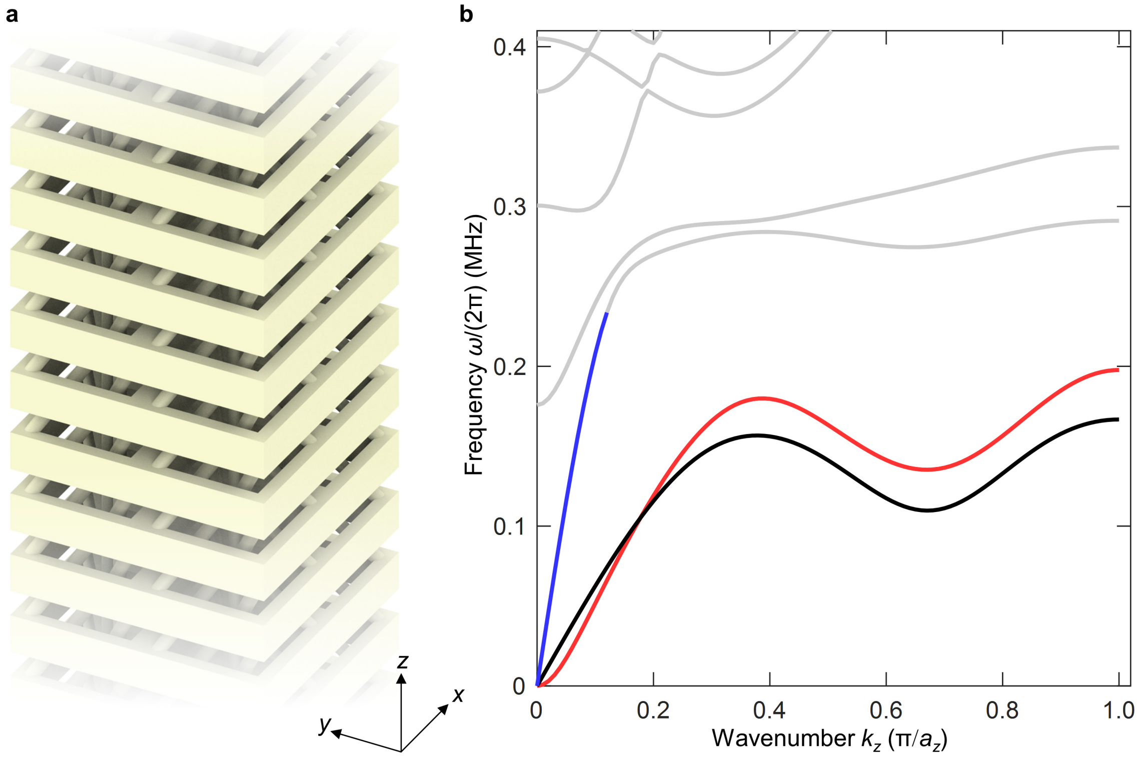


**Supplementary Figure 4 | Phonon band structure of an elastic metamaterial beam.** (a) We consider a beam with a cross section of merely 2 × 2 unit cells as shown in Fig. 3(a). The beam is periodic along the $z$-direction. (b) Corresponding band structure $\omega(k_z) = \omega(-k_z)$ for propagation of elastic waves along the $z$-direction with wavenumber $k_z$. The two degenerate flexural or transverse-like bands (red) exhibit a roton-like behavior analogous to that of the two degenerate transverse bands in the bulk (cf. red curve in Fig. 4). As usual, the flexural bands start quadratically versus $k_z$ for beams with finite cross section. The longitudinal band (blue) exhibits an avoided crossing with low-lying "optical" bands (gray), obscuring the roton-like behavior. The twist band (black) only occurs for metamaterial beams with finite cross section. It does not exist in the bulk (cf. Fig. 4). This twist band also features a roton-like behavior.

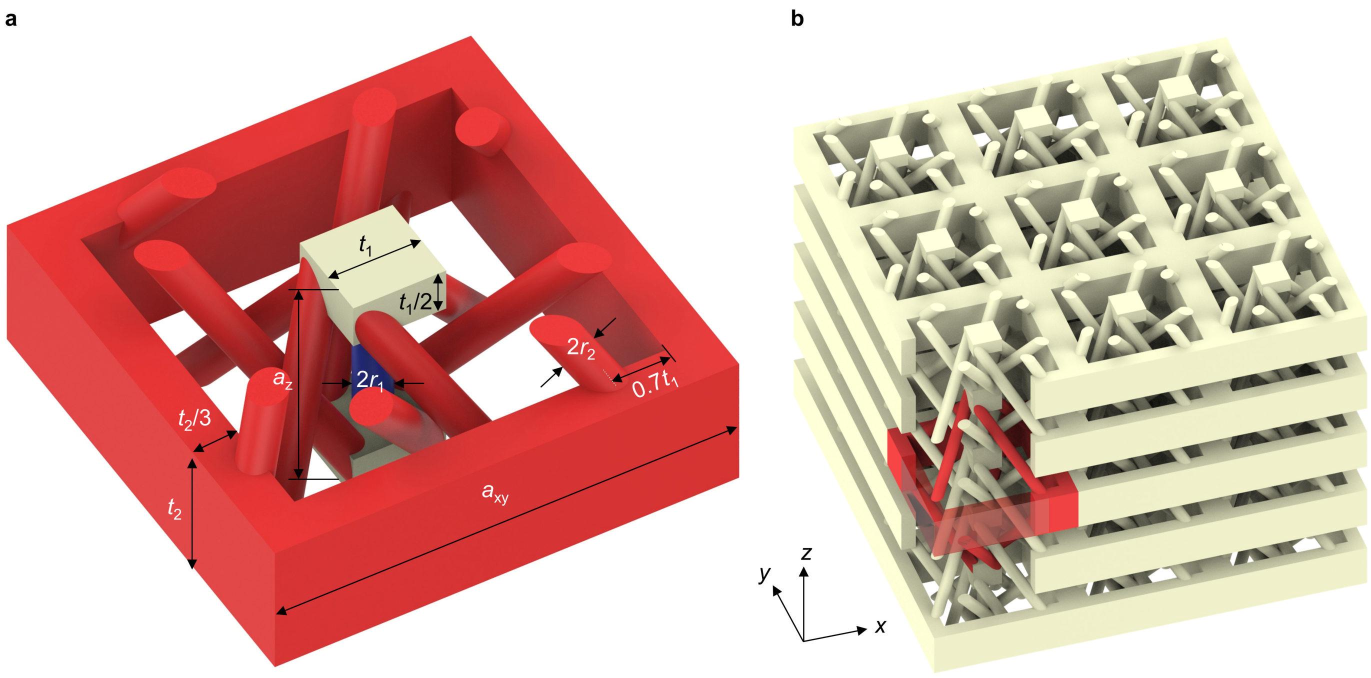


**Supplementary Figure 5 | Designed three-dimensional chiral elastic metamaterial structure.** As Fig. 3 (achiral), but the oblique rods are “twisted”, making the structure chiral. In addition, the number of these rods is doubled to obtain four-fold rotational symmetry around the $z$-axis. Geometric parameters are the same as those in the previous achiral design (cf. Fig. 3(a)) except for $r_2/a_z = 0.08$. (a) Oblique view onto a single unit cell. (b) $3 \times 3 \times 5$ unit cells out of a corresponding bulk metamaterial.

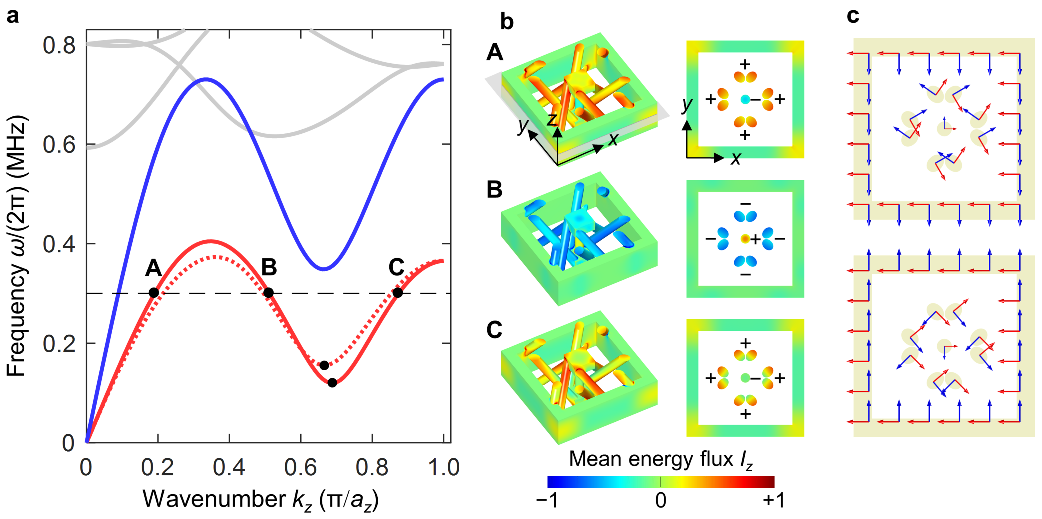


**Supplementary Figure 6 | Chiral elastic metamaterial phonon band structure.** (a) phonon band structure as in Fig. 4(a), but for the chiral architecture shown in Supplementary Figure 5. The degeneracy of the two transverse bands (red) is lifted due to chirality. One handedness is shown by the solid curve, the other by the dashed curve. (b) Mean energy flux $I_z$ along the $z$-direction (on a false-color scale) corresponding to three eigenmodes marked as **A**, **B**, and **C** for the same frequency of the red solid transverse band. (c) For the two black dots in the two roton minima of the dispersion relations, we show the real (blue) and imaginary (red) part of the local displacement vector within the $xy$-plane. If the real and imaginary part are perpendicular to each other and have the same length, the displacement vector moves in circles around its rest position. This behavior indicates rotations associated to chiral phonons.
