# Supplementary material for: Roton-like acoustical dispersion relations in 3D metamaterials
Source: Nat Commun. 2021 Jun 2;12:3278. doi: 10.1038/s41467-021-23574-2 (PMC8172548; doi:10.1038/s41467-021-23574-2)
Supplement: Supplementary file 3 — Description of Additional Supplementary Files [file 41467_2021_23574_MOESM3_ESM.pdf]

## Description of Additional Supplementary Files

**Supplementary Movie 1:** An animated view of the structure in Fig. 3(b)
