# Supplementary figures and images for: Roton-like acoustical dispersion relations in 3D metamaterials
Source: Nat Commun. 2021 Jun 2;12:3278. doi: 10.1038/s41467-021-23574-2 (PMC8172548; doi:10.1038/s41467-021-23574-2)

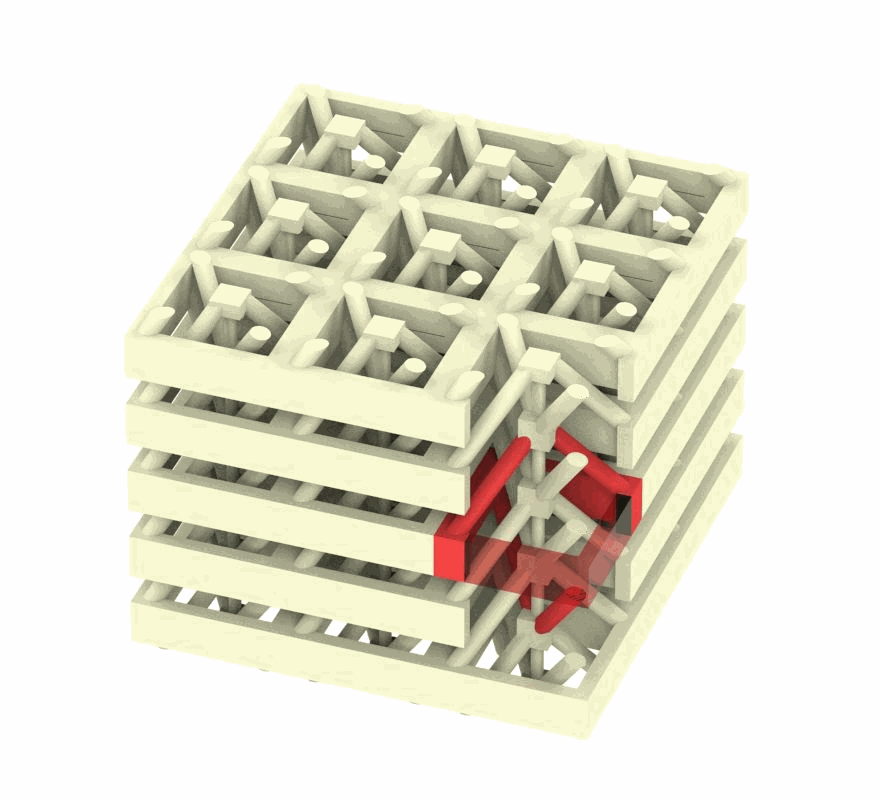

Supplement: Supplementary file 4 — Supplementary Movie 1 [file 41467_2021_23574_MOESM4_ESM.gif]
